# Trace Elements in the Large Population-Based HUNT3 Survey

**DOI:** 10.1007/s12011-020-02376-5

**Published:** 2020-09-08

**Authors:** Tore Syversen, Lars Evje, Susann Wolf, Trond Peder Flaten, Syverin Lierhagen, Anica Simic

**Affiliations:** 1 Department of Neuromedicine and Movement Science, Faculty of Medicine and Health Sciences, NTNU, Norwegian University of Science and Technology, N-7491 Trondheim, Norway; 2 Present Address: Department of Earth Science, Faculty of Mathematics and Natural Science, University of Bergen, N-5007 Bergen, Norway; 3 Present Address: National Institute of Occupational Health, N-0363 Oslo, Norway; 4 Department of Chemistry, Faculty of Natural Sciences, NTNU, Norwegian University of Science and Technology, N-7491 Trondheim, Norway

**Keywords:** Population-based study, Trace elements, ICP-MS analysis, Blood samples

## Abstract

The Nord-Trøndelag Health Study (The HUNT Study) is a large health survey population study in the county of Trøndelag, Norway. The survey has been repeated four times in about 10-year intervals. In the HUNT3 survey (2006–2008), we collected 28,000 samples for trace element analysis. Blood samples from 758 healthy persons without known occupational exposure were selected for multielement analysis of a small sample of blood (0.25 mL). The aim of the study was to determine the minimum blood volume that can be used for the analytical procedure and to compare our results with previously published results of similar surveys in healthy populations. Samples were digested and the concentration of selected trace elements was determined by ICP-MS. We report results on essential elements (B, Co, Cu, Mn, Se and Zn) as well as non-essential elements (As, Be, Br, Cd, Cs, In, La, Pb, Hg, Nd, Ni, Nb, Pd, Pt, Sm, Ta and Sn). Results are similar to previous studies on the HUNT3 population, and with a few exceptions, our data compares very well with results obtained in recent studies from other countries. We wanted to test a minimum volume of blood in a large-scale analytical program. For a number of nonessential elements, our results were below the limit of detection. We suggest that future studies using similar ICP-MS equipment as analytical tool should use at least 0.5 mL of blood.

## Introduction

It is a fundamental principle in pharmacology and toxicology that the effect of an agent becomes more likely if the dose or exposure increases. Most environmental contaminants that may induce health effects behave this way. For trace elements, it becomes somewhat more complicated. The basic principle may apply when we consider non-essential elements while we for essential elements may also observe poor health when the exposure is below a minimum requirement for that organism. Apart from individual differences in genetic character, it is our environment that is most likely to influence our body’s trace element composition. Inadequate body burden of essential elements can be caused by the diet being deficient in the particular element or that other factors in the local environment may change the uptake efficiency of that essential element [1]. Thus, when we correlate health parameters and trace elements in, e.g. blood, we cannot readily determine whether health changes are the result of changes in, e.g. blood levels of that particular trace element—or if the body burden of that element has changed as a result of, for example, exposure to some other element. To complicate the issue further, we do know that a disease may, by itself or through necessary medication, change trace element metabolism. Thus, when we find a correlation between a single time point blood sample and a health indicator, we should always ask ourselves—is it the change in trace element body burden that induces the health effect or the other way around that the health condition has caused changes in trace element metabolism. One approach to resolve such challenges is to medically examine and sample the same population repeatedly over an extended time period. This is one aim for our HUNT Survey of trace elements and health.

The HUNT Survey in Norway is a large population-based survey consisting of cohorts from HUNT1 (1984–86), HUNT2 (1995–1997) and HUNT3 (2006–2008). The HUNT4 survey started in 2017. Originally, the HUNT Survey was primarily set up to examine arterial hypertension, diabetes, tuberculosis screening and quality of life. The later surveys have included an increasing number of health parameters and the HUNT3 survey includes a modern biobank. This biobank includes blood samples for several purposes. Included are about 28,000 samples of 7 mL whole blood collected on test tubes designed for trace element analysis. This 7 mL sample was on arrival at the biobank divided into several 0.7 mL aliquots and then stored at − 80 °C or in liquid nitrogen. These samples are available for analysis of trace elements as well as environmental contaminants. More details on the HUNT studies can be found on their website [2] and in the overview presented by Krokstad et al. [3].

A prime interest to us has been the possibility to access health data recorded decades before the blood sample was collected—as well as collecting a blood sample that can be stored and compared with future samples from the same person. As analytical equipment and techniques develop, it is very important to store samples in such a way that they can be accessed and used for chemical analysis in the future. The HUNT facility provides such safe storage and also makes analytical data available that has been collected on the samples. We have already published two studies on diabetes and trace elements [4, 5]. We now report data for a cohort of some 757 participants in the HUNT3 Survey using only a 0.25 mL blood sample. This cohort was adopted from another survey (HUNT-NMR, see, e.g. [6–8]) and includes a healthy normal population with a limited age range living in the same area in Norway. Our main objective in the present survey was to develop and test procedures for processing, analysing and documenting large numbers of small blood samples for trace element analysis. As a result of this, we here present reference values for some trace elements in a healthy and medically well-documented population.

## Methods

### The HUNT3 Biobank, Sampling and Storage

Trace element samples for the HUNT biobank were collected from 14 municipalities ranging between 460 and 15,498 inhabitants. A total of 28,084 persons above the age of 13 were sampled using standard blood venipuncture equipment (Vacuette, Greiner Bio-One North America, Inc., Monroe, NC) to collect 7 mL of blood in trace element vacutainer tubes. For part of the survey presented here, samples were collected using Na-heparin as an anticoagulant (Vacutainer Cat. no. 367735; Becton, Dickinson & Co, Franklin Lakes, NJ). The trace element sample tube was the last of five vacutainers filled at the same time. This precaution was taken in order to flush the needle used and thus reduce any contamination of trace elements from the sampling equipment. The samples were kept refrigerated and on the same day transported to the biobank facility at Levanger (Norway). Each sample was then divided into seven 0.7 mL samples kept in polyethylene sample tubes (Thermo Scientific). Six of these are kept at − 80 °C and one in liquid nitrogen*.*

### Cohort

The population selected for the present survey was a cohort originally used for a population neuroimaging survey. The selection criteria were age between 50 and 65 years and that they had participated in the earlier HUNT surveys (HUNT1 1984–1986 and HUNT2 1995–1997). Exclusion criteria were limited to MRI contraindications: pacemaker of the heart, clipped cerebral aneurysm, cochlear implants, severe claustrophobia and body weight above 150 kg. There were 1006 participants in the neuroimaging survey, and a detailed epidemiological description of this population has been published [9]. In this population (1006 participants), blood samples had been collected for 758 persons. Table 1 shows that our survey population is equally divided between men and women. The average age is 57.4 years with a range of 49–66 years. Body mass index (BMI) is on average 27 (range: 18–42). Smoking compares well with the Norwegian national survey (21% smokers average for both genders).

**Table 1:** Main demographic characteristics of the examined group (*N* = 757). For the mean waist-to-hip ratio, the mean and range is given. For more details on the cohort, see [9]

Characteristics	Total (*n* = 757)	Men (*n* = 375)	Women (*n* = 382)
Age (years, SD)	57.4 (4.2)	57.6 (4.2)	57.3 (4.2)
BMI (kg/m^2^, SD)	27.0 (3.6)	27.4 (3.1)	26.7 (4.0)
Mean waist-to-hip ratio	0.90 (0.68–1.17)	0.94 (0.06)	0.87 (0.06)
Smoking, never (*n*, %)	263 (35.3)	134 (36.2)	129 (34.4)
Smoking, former (*n*, %)	324 (43.5)	170 (49.0)	154 (41.1)
Smoking, current (*n*, %)	158 (21.2)	66 (17.8)	92 (24.5)
Occupationally active (*n*, %)	628 (82.9)	321 (85.6)	307 (80.4)

### Analysis

A 0.25 mL blood aliquot from the samples and certified reference material (Seronorm Trace Element Whole Blood L-1, LOT MR 4206, Sero, Norway) was transferred to a preweighed 8 mL PTFE digestion vessel. The weight was recorded, and the accurate sample mass was thus obtained. As an internal standard, 0.500 mL of 12 μg/L rhenium in 32.5% HNO_3_ was added. A typical digestion run included 67 samples, 5 blanks and 5 Seronorm-certified reference material. Samples were digested in an UltraCLAVE microwave digestion system (Milestone, Italy) using a program of incrementing the temperature from 20 to 245 °C in 60 min and left at 245 °C for 10 min. The samples were left in the digestion tubes until the next day in a clean air cabinet. Then 5.25 mL of ultrapure water was added. The sample was thoroughly mixed and decanted into a pre-washed polypropylene tube and stored at 4 °C until analysed.

The samples were at room temperature at the time of ICP-MS analysis (Element 2, Thermo Finnigan, Bremen, Germany). The radio frequency power was set at 1350 W.

The samples were introduced using an SC-E2 autosampler with SC-FAST option (ESI, USA) with a peristaltic pump (0.2 mL/min).

The instrument was equipped with a concentric PFA MicroFlow nebuliser (ESI, USA) connected to a Scott PFA spray chamber (ESI), aluminium X skimmer and sampler cones (ESI) with demountable quartz torch. The nebuliser argon gas flow rate was adjusted to give a stable signal with maximum intensity for the nuclides ^7^Li, ^115^In and ^238^U. Ten percent methane in argon was added to the argon plasma at the front of the spray chamber at an approximate flow of 20 mL/min. The instrument was calibrated using 0.6 M HNO_3_ solution of matrix-matched multielement standards. A calibration curve using 5 different concentrations was made from these standards (M-320, M-321 and M-322, SpectraPure, Norway). To check for instrumental drift, a multielement standard (High-Purity Standards, USA) were analysed for every 10 samples. Certified reference material was analysed at the start and end of each analytical sequence.

The accuracy of the method was verified by repeated analyses of the certified reference materials Seronorm whole blood and Seronorm Serum Level 1. The concentrations found were within 85–115% of the certified values. The precision of the method was checked by analysing four separate preparations of the same samples and it was better than 15% relative standard deviation for all elements.

### Selection of Resolution and Isotopes for some Elements

Boron (Be11) was determined in low resolution (LR) and medium resolution (MR). Doubly charged (Na++) can interfere with the boron measurement in LR. As results obtained in LR and HR were the same, we selected the LR results as the instrumental relative standard deviation (RSD) was less.

Selenium was determined in LR (Se82) and HR (Se78). Se78 in HR is free of interferences. However, comparing the results for Se82 (LR) and Se78 (HR) identical results and LR gave better instrumental RSD and was therefore chosen.

Both Cd111 and Cd114 were determined in MR. Cd114 was chosen because of higher abundance and better baseline stability. The isobaric overlap from Sn was corrected using the correction equation in the instrument software.

### Statistical Analysis

Descriptive statistics were generated using STATA 13 software (StataCorp, TX).

## Results

Due to the limited blood volume available, we were able to analyse whole blood only. The data obtained are presented in Table 2. For several elements reported in our study, serum or plasma is primarily selected for analysis. Thus, there are not many studies in the recent literature to which we can compare the whole blood values obtained in our present study. In Table 3, we have compared our data with some recent reports in the literature.

**Table 2:** Trace elements in blood (μg/L). A total of 757 samples were analysed. The column marked isotope gives the isotope used to measure the element and resolution indicate the instrumental resolution set for analysis. LOD is limit of detection

	Symbol	<LOD (%)	Isotope	Resolution	LOD	Mean	Min	Max	Median	10%	25%	75%	90%
Essential elements													
Boron	B	2.8	11	LR	0.50	25.58	< 0.50	154.58	21.91	6.82	13.05	32.67	46.85
Cobalt	Co	11.8	59	MR	0.040	0.137	< 0.040	1.053	0.104	< 0.040	0.063	0.175	0.271
Copper	Cu	–	63	MR	0.30	1078	676	1837	1040	865	938	1178	1366
Manganese	Mn	–	55	MR	0.06	9.62	3.96	29.74	9.12	6.07	7.39	11.32	13.77
Selenium	Se	–	82	LR	0.50	115.4	60.6	201.2	112.7	90.5	98.9	129.1	145.1
Zinc	Zn	–	67	MR	0.40	8085	4424	17,152	7629	6253	6791	8829	10,835
Nonessential elements													
Arsenic	As	1.6	75	HR	0.25	4.16	< 0.25	32.9	2.88	0.89	1.54	5.03	8.84
Beryllium	Be	55.6	9	LR	0.020	0.035	< 0.020	0.359	< 0.020	< 0.020	< 0.020	0.047	0.088
Bromine	Br	–	81	HR	30.0	2004	494	11,319	1914	1174	1549	2362	2794
Cadmium	Cd	3.4	114	MR	0.100	0.548	< 0.100	3.416	0.355	0.154	0.224	0.583	1.212
Caesium	Cs	–	133	LR	0.005	5.32	1.89	19.6	5.03	3.63	4.19	5.99	7.23
Indium	In	0.8	115	LR	0.005	0.089	< 0.005	0.367	0.082	0.033	0.059	0.113	0.154
Lanthanum	La	17.6	139	MR	0.020	0.050	< 0.020	0.226	0.055	< 0.020	0.032	0.065	0.081
Lead	Pb	–	208	LR	0.02	23.81	5.25	128.7	21.10	12.59	16.02	27.73	37.08
Mercury	Hg	–	202	LR	0.01	3.57	0.22	20.79	2.75	1.35	1.95	4.19	6.51
Neodymium	Nd	0.7	146	LR	0.002	0.032	< 0.002	0.137	0.032	0.012	0.021	0.042	0.051
Nickel	Ni	12.5	60	MR	0.15	0.77	< 0.15	6.17	0.52	<0.15	0.26	0.97	1.63
Platinum	Pt	69.6	195	LR	0.010	0.011	< 0.010	0.170	<0.010	<0.010	<0.010	0.012	0.024
Samarium	Sm	60.9	147	LR	0.005	0.006	< 0.005	0.058	<0.005	<0.005	<0.005	0.009	0.013
Tantalum	Ta	52.6	181	LR	0.002	0.003	< 0.002	0.024	0.002	<0.002	<0.002	0.004	0.006
Tin	Sn	58.1	118	LR	0.040	0.206	< 0.040	2.714	<0.040	<0.040	<0.040	<0.160	0.501

**Table 3:** Comparison of element concentrations (μg/L) in whole blood in the present study with those of some recently published studies of adults. Values are given in μg/L and as geometric means (GM) or medians as indicated in the column headings

	Symbol	Present study	Hansen [4] 2017	Simic [5] 2017	Heitland [10] 2006	Alimonti [11] 2011	Schultze [12] 2014	Nisse [13] 2017	Snoj Tratnik [14] 2019
Country		Norway	Norway	Norway	Germany	Italy	Sweden	France	Slovenia
Samples		757	755		130	1423	1000	1992	1084
Value		Median	Median		GM	GM	Median	GM	Median
Essential									
Boron	B	21.91	27.5	27.69	36				
Cobalt	Co	0.104			0.14	0.147		0.30	
Copper	Cu	1040	1010	1008	1020		813		940
Manganese	Mn	9.12	9.1	8.93	8.6	8.2	7.58	7.7	13.3
Selenium	Se	112.7	101.4	99.07	132				104
Zinc	Zn	7629	7540	7510			6278	5805	6636
Nonessential									
Arsenic	As	2.88	2.90	2.55	0.71	1.14		1.67	0.77
Beryllium	Be	< 0.020			< 0.008	0.085		0.003	
Bromine	Br	1914	1560	1533					
Cadmium	Cd	0.355	0.35	0.31	0.38	0.53	0.27	0.39	0.29
Caesium	Cs	5.03	4.60	4.38	3.4				
Indium	In	0.082	0.028	0.029	< 0.009				
Lanthanum	La	0.055			< 0.008				
Lead	Pb	21.10	19.9	18.42	19	19.9	17.2	18.8	17.5
Mercury	Hg	2.75	3.18	2.73	0.9	1.19	1.79	1.38	1.20
Neodymium	Nd	0.032							
Nickel	Ni	0.52	0.49	0.50	0.08	0.89	0.53	1.31	
Platinum	Pt	< 0.010				0.014			
Samarium	Sm	< 0.005							
Tantalum	Ta	0.002	0.0026	0.0025					
Tin	Sn	< 0.040	0.19	0.20	0.12	0.54			

For ease of presentation and discussion, we have divided the elements into two groups: essential and non-essential. The assignment as essential is in some cases an issue of debate, and we have restricted the term “essential” only to elements where a known biological function in mammalians has been determined [15].

### Essential Elements

For boron and cobalt, many samples were below LOD (limit of detection). The present data for the essential elements compares very well with previously published data from two other populations from the same HUNT3 Survey [4, 5] Further details can be found in Table 3.

### Nonessential Elements

We analysed a very small blood sample. Sample treatment was designed to cause minimum dilution of the sample. From Table 2, we can see that a number of element concentrations were found to be below the detection limit. For the important toxic elements like arsenic, cadmium, lead, mercury and nickel, the small sample volume can be used. For the typical ultra-trace elements, a larger blood volume has to be used, typical examples being beryllium, platinum and tin.

## Discussion

The biochemistry and toxicology of the analysed elements are beyond the scope of this paper; the reader is referred to a vast literature on these aspects. The discussion is thus focused on comparing the present data with recent similar population studies.

In Table 3, we have selected some recent population studies in various European countries: Germany [10], Italy [11], Sweden [12], France [13] and Slovenia [16]. For the essential elements, we would expect some variations between countries as diet and environment may cause regional differences. This is reflected in the levels reported for, e.g. copper, manganese, selenium and zinc. For boron, we find lower values than Heitland (2006) in Germany and the same for cobalt in France [13]. Even higher values for boron have been found by Koc et al. [17]. They reported values of 56 ± 50 μg/L, which is twice as much of what we have found in the three HUNT3 populations studied so far. For cobalt, a normal blood level of 0.5 μg/L has been suggested [18]. We found 0.104 μg/L in the present study.

Although copper in blood is metabolically well controlled, it certainly does differ in various population studies. Our median value 1040 μg/L compares well with the previous values from the HUNT3 Survey; 1010 [4]; 1008 [5] as well as 1020 in a German study [10].

Whole blood levels of manganese may vary considerably (7–12 μg/L) and seem to reflect environmental exposure through food, water and air [19]. In a large survey of 350 people using neutron activation analysis, a value of 8.8 μg/L was found [20]. In the present survey, we found 9.12 μg/L which is similar to the other HUNT3 studies by Hansen [4] and Simic [5] as well as Heitland [10]. These are all values that we would expect to be found in persons without occupational exposure.

In our population, we found about 10% higher selenium concentration in blood compared with the other two HUNT3 populations that have been examined [4, 5]. As fish consumption will vary within the larger HUNT3 population, such a difference is not surprising. All these values are within the range of the other studies listed in Table 3.

Zinc blood content depends to a large extent on the diet and shows very similar values for the three HUNT3 populations and compares well with reports from other parts of Europe (Table 3).

### Non-essential Elements

None of the HUNT3 participants in the present study have reported occupational exposure to any of the non-essential elements included in our study. Nevertheless, when we compare blood levels of these elements in different regions, we will find differences arising from environmental exposure, e.g. air, water and diet.

Seafood is known to contain large amounts of arsenic compounds, and thus a marine diet may greatly influence both blood and urinary levels of arsenic. Concha et al. [21] studied children and women with high arsenic exposure and reported a median value of 0.95 μg/L (0.69–1.8) for the controls (occupationally non-exposed population). The HUNT3 populations show median values of 2.55–2.90 μg/L, while other studies in Norway have shown values of 1.46 μg/L (21) and 5.9 μg/L [22]. A survey from Germany [10] showed a much lower value of 0.71 μg/L. The higher levels reported from the HUNT3 participants may reflect that pelagic fish is an important part of the diet in this part of Norway.

Beryllium exposure is by far dominated by air contamination in an industrial setting. The element is poorly absorbed from the intestine, and the level we found is in between those observed in Germany [10], Italy [11] and France [13]. It should be noted that, for our population, 55% of the results were below the level of detection.

The cadmium levels found in the three HUNT3 populations compare well with the findings in other European studies (see Table 3 for details). Only Italy found elevated levels, however, well below several other studies in occupationally non-exposed populations. Byber et al. [23] have recently published an extensive review.

Caesium levels are similar in all three HUNT3 populations and higher than the findings in Germany [10].

Indium is an important component in semi-conductors and thus reaches our environment through a range of electronic products we use every day. The toxicology of indium tin oxide related with occupational exposure has recently been reviewed [24]. We have found a range of values (0.028–0.082) in our HUNT3 populations, while Heitland reported < 0.009 μg/L in their German population [10].

Lanthanum is used in a number of products including catalysts, electronics and glass additives. The blood level recorded in our survey (0.055 μg/L) is far higher than that reported by Heitland et al. [10].

Lead was used as an additive in gasoline and for many decades airborne lead particles were distributed worldwide and eventually ended up in the food chain. Many countries introduced restrictions on leaded gasoline from the 1970s and lead is now banned as a gasoline additive in most countries. Thus, it is not surprising that Minoia report a value of 157 μg/L in their 1990 report [20] and we now see a range of values 7.4–24.5. Blood levels of lead are expected to become even lower in the coming years.

Freshwater and some saltwater fish will contribute to human exposure to organic mercury, in particular methylmercury that is extremely well absorbed from the intestine. The other main source (mercury vapour) released from amalgam fillings in teeth. The age group addressed in our survey will have amalgam fillings and freshwater fish is readily available. We can see that European populations with less fish in the diet than in Norway do have a lower blood mercury content (see Table 3). However, the blood mercury level observed here does not imply any reason for alarm; see review by Syversen and Kaur [25] .

Neodymium belongs to the rare earth elements. It is used as a glass dye and in the production of very strong permanent magnets. It is used in many consumer products that may eventually end up in our trash and then become widely distributed in nature. Very little is known about its toxicology, and the most recent account of general toxicology we could find is from 1964 [26]. We found a mean value of 0.032 μg/L which is much lower than the mean value of 1.39 μg/L reported by Minoia et al. [20]. Based on its widespread use in, e.g. consumer electronics, the environmental impact of neodymium should be considered more closely.

Most biomonitoring population studies of nickel have analysed urine or serum/plasma. However, a few recent studies of blood levels are included in Table 3. We see that our three HUNT3 studies compare very well, while the other European studies vary considerably. When comparing various studies, careful attention should be made towards sampling techniques as well as analytical procedures. Low-level samples of nickel are easily contaminated.

For platinum, samarium, tantalum and tin, more than 50% of the samples had blood levels below the detection limit. We still report the values obtained here, but for these elements, it is obvious that a larger blood sample volume is required in order to obtain trustworthy values.

## Concluding Remarks

We used a small blood sample (0.25 mL) for analysis, and this is a challenge as many measurements become very close or below the detection level of the instrumental analysis. The results will be liable to many types of errors: contamination during sample collection, storage and handling; laboratory environment, equipment and chemicals; and instrumental stability.

## Data Availability

A complete copy of data has been deposited in the databank of HUNT/NTNU.
